# A92 THE CURRENT STATE OF PEDIATRIC TO ADULT TRANSITION OF CARE: A ROUND TABLE DISCUSSION WITH KEY STAKEHOLDER GROUPS

**DOI:** 10.1093/jcag/gwad061.092

**Published:** 2024-02-14

**Authors:** M Hu, N Gakhal, C Kabakulak, J LoMonaco, N Bollegala

**Affiliations:** University of Toronto Temerty Faculty of Medicine, Toronto, ON, Canada; University of Toronto Temerty Faculty of Medicine, Toronto, ON, Canada; Women's College Hospital, Toronto, ON, Canada; Women's College Hospital, Toronto, ON, Canada; University of Toronto Temerty Faculty of Medicine, Toronto, ON, Canada

## Abstract

**Background:**

There is a severe paucity in available resources to facilitate a safe and effective pediatric to adult health care transition for patients with chronic disease.

**Aims:**

This study aims to better understand the experiences of key stakeholder groups involved in pediatric to adult transition of care across various chronic disease specialties within a tertiary care urban environment.

**Methods:**

A focus group was conducted on July 12th, 2018 at Women’s College Hospital with key stakeholder groups. Questions focused on the top issues related to pediatric to adult transition of care and an ideal transition model for their respective patient populations. The second part of this study included individual interviews with patients and carepartners. Focus group meetings and individual interviews were both audio-recorded and transcribed for qualitative thematic analysis.

**Results:**

Seventeen pediatric and adult care physicians representing ten chronic disease specialties were represented. Seven patients and care partners were interviewed. Major focus group themes included: disproportionate access to resources between and within specialties (n=10), lack of mental health support in the adult setting (n=8), fragmentation of care due to multiple providers as a result of patients leaving for employment or education (n=6), patients feeling alienated due to decrease in allied health support or due to shorter appointment times (n=8), and difficulty retaining consistent care due to missed appointments as a result of inconsistent communications or change in location for education or employment (n=7). Ideal care models were suggested to include: centralized intake procedure to identify patients who may require extra support (n=9), education for scheduling administrators about second chances after a no-show (n=5), implementation of joint clinics with pediatric and adult care teams or back-and-forth clinic sessions to ensure bidirectional provider comfort before discharge to adult care system (n=4), and implementation of a dedicated transitions navigator or point of contact for patients (n=10).

The individual patient and carepartner interviews emphasized the need for: centralized and comprehensive online resource (n=7), simple and clear educational content introducing the adult healthcare system (n=7), opportunities to meet and share information with other individuals in a similar position (n=3), and availability of a transitions navigator (n=4).

**Conclusions:**

Current pediatric to adult transition of care resources are fragmented, allocated inefficiently, difficult to ascertain and often constrained to a specific condition or location. Future efforts should focus on improving the care transition process for high-risk patient populations by taking a comprehensive approach utilizing innovative, and multidisciplinary solutions.

**Funding Agencies:**

None

